# Development and Validation of the Autism Behavior Assessment Scale (ABAS)

**DOI:** 10.3390/children12081038

**Published:** 2025-08-08

**Authors:** Ibrahim Halil Diken, Ozlem Diken, Umit Isik

**Affiliations:** 1 Research Institute for Individuals with Developmental Disabilities, Anadolu University, Tepebaşı 26470, Türkiye; 2 Research, Education and Training Center for Individuals with Speech and Language Disorders, Anadolu University, Tepebaşı 26470, Türkiye; odogramaci@anadolu.edu.tr; 3 Umit Isik Academy, Child and Adolescent Psychiatry, Isparta, Türkiye; crsumt@gmail.com

**Keywords:** Autism Spectrum Disorder, assessment, psychometric validation, scale development, Türkiye, ABAS

## Abstract

**Background**: Autism Spectrum Disorder (ASD) encompasses a range of neurodevelopmental conditions characterized by impairments in social communication, restricted and repetitive behaviors, and sensory sensitivities. Despite increased awareness, timely diagnosis in Türkiye remains limited due to the lack of culturally appropriate, psychometrically robust assessment tools. **Objective**: This study aimed to develop, validate, and standardize the Autism Behavior Assessment Scale (ABAS) as a reliable and culturally adapted tool for assessing ASD-related behaviors in individuals aged 3–24 years in Türkiye. **Methods**: Employing a three-phase, nine-step scale development framework, data were gathered from 1275 informants (parents and professionals) across 14 provinces. The ABAS comprises 36 items rated on a three-point Likert scale, spanning four subscales: Restricted Repetitive Behaviors & Sensory Sensitivity (RRBSS), Social Interaction (SI), Social Communication (SC), and Non-Developmental Speech (NDS). Psychometric analyses included exploratory and confirmatory factor analysis, reliability testing, and validation against established instruments. **Results**: The four-factor structure was confirmed via EFA and CFA with excellent model fit. The ABAS demonstrated strong internal consistency (α = 0.91–0.96), test–retest reliability (r = 0.83), and criterion validity (r = 0.93 with GARS-2-TV; r = 0.84 with U-ODKL). Discriminant validity analyses showed that the ABAS accurately differentiated individuals with ASD from individuals with intellectual disabilities (ID) and individuals with hearing impairments (AUC = 0.99). **Conclusions**: The ABAS is a psychometrically sound, developmentally sensitive, and culturally grounded instrument for identifying and monitoring ASD-related behaviors in Türkiye. It holds promise for improving early detection and guiding educational and clinical interventions.

## 1. Introduction

Autism Spectrum Disorder (ASD) is defined by the Diagnostic and Statistical Manual of Mental Disorders, Fifth Edition, Text Revision (DSM-5-TR) as a neurodevelopmental condition with persistent deficits in social communication and social interaction, along with restricted, repetitive patterns of behavior, interests, or activities [1]. These deficits manifest early in development and significantly impact an individual’s daily life. DSM-5-TR highlights the heterogeneity of ASD, recognizing that symptoms vary widely in presentation and severity. The two core diagnostic criteria for ASD in DSM-5-TR are persistent deficits in social communication and interaction, including challenges in reciprocal social interactions, difficulty in understanding and using nonverbal communication, problems in developing, maintaining, and understanding relationships, and restricted, repetitive patterns of behavior, interests, or activities, including stereotyped movements, insistence on sameness, highly fixated interests, and unusual sensory responses.

ASD is now considered a spectrum due to its varied manifestations and severity levels, requiring individualized assessments to determine support needs [2]. Early diagnosis of ASD is crucial for improving long-term outcomes, as early intervention can significantly enhance cognitive, social, and adaptive functioning. Research suggests that ASD symptoms may emerge as early as 12–18 months of age, with common early indicators including reduced eye contact, lack of response to name, and delayed speech development [3]. The ability to identify ASD early allows for timely interventions, which have been shown to improve communication skills, social interaction, and behavioral regulation [4].

Behavioral markers serve as critical indicators in the assessment and diagnosis of ASD, offering observable and measurable manifestations of the core symptoms defined in diagnostic manuals such as the DSM-5-TR. Unlike purely cognitive or physiological assessments, behavioral markers allow for direct observation across naturalistic, clinical, and structured contexts, making them indispensable in both early detection and differential diagnosis processes. One of the most consistently reported domains is impairment in social communication, which encompasses a range of behaviors that deviate from typical developmental trajectories. Individuals with ASD often exhibit reduced eye contact, limited joint attention, and difficulties in interpreting nonverbal cues such as facial expressions and gestures [2,5]. These deficits hinder reciprocal social interactions, leading to challenges in forming and maintaining peer relationships. Pragmatic language difficulties—such as an inability to appropriately take turns in conversation, literal interpretation of language, and difficulty understanding idioms or sarcasm—are also prevalent [6]. In addition to social communication difficulties, repetitive behaviors and restricted interests are hallmark features of ASD. These include stereotyped motor movements such as hand flapping, body rocking, or spinning, as well as verbal repetitions like echolalia. Many individuals with ASD also display a strong insistence on sameness, rigid adherence to routines, and intense preoccupations with narrow topics, often with encyclopedic knowledge in those areas [7,8]. Such behaviors, while sometimes adaptive or self-regulatory, are often inflexible and interfere with daily functioning and learning. These traits have been found to persist across the lifespan and remain a central axis in both diagnosis and treatment planning [9].

A growing body of research also emphasizes the importance of sensory processing abnormalities as a behavioral marker for ASD. Sensory sensitivities are now included in the DSM-5-TR diagnostic criteria, reflecting their near-universal presence among individuals with ASD [10]. These sensitivities can manifest as over-responsiveness—such as aversion to loud sounds, bright lights, or certain textures—or under-responsiveness, such as indifference to pain or temperature. Some individuals with ASD display unusual sensory-seeking behaviors, including fascination with light patterns or repetitive visual inspection of objects. Empirical studies report that over 80% of individuals with ASD experience sensory processing difficulties that significantly affect their participation in daily life activities [11,12]. Another critical domain involves atypical speech and language patterns, which can include delayed speech onset, reduced expressive language, or the use of unconventional language forms. Nonfunctional speech characteristics—such as echolalia, scripting, idiosyncratic phrases, or unusual prosody—are frequently observed [13,14]. These features often coexist with other communication impairments, limiting an individual’s ability to engage in typical social exchanges. Furthermore, studies suggest that early speech patterns can serve as predictors for later developmental outcomes, emphasizing their diagnostic relevance [15].

Collectively, these behavioral markers—spanning social communication, restricted and repetitive behaviors, sensory sensitivities, and speech anomalies—form the core dimensions for ASD assessment. Standardized observational tools, such as the Autism Diagnostic Observation Schedule (ADOS) and the current tool, the Autism Behavior Assessment System (ABAS), often rely heavily on these markers for clinical scoring and interpretation. As such, the identification and evaluation of these behaviors must be grounded in developmental norms, culturally sensitive practices, and multidisciplinary input. Enhanced training of professionals to recognize nuanced behavioral presentations—especially in underdiagnosed populations such as girls, culturally diverse groups, and individuals with high-functioning profiles—remains a key priority in advancing early and accurate ASD diagnosis.

The prevalence of ASD has increased globally, with the Centers for Disease Control and Prevention (CDC) reporting that as of 2022, approximately 1 in 31 (3.2%) children aged 8 years have been identified with ASD, up from 1 in 36 (2.8%) in 2020 and 1 in 150 in 2000 [16]. National data on ASD prevalence in Türkiye remain limited and fragmented. However, extrapolating from global estimates and small-scale epidemiological studies suggests a growing need for structured identification and support systems. For example, according to a population projection by the Turkish Statistical Institute [17], an estimated 1.3 million individuals in Türkiye may be on the autism spectrum. Similar trends have been observed worldwide, indicating a growing need for efficient diagnostic frameworks. Studies on autism prevalence in Türkiye are limited.

In recent years, the assessment and diagnosis of individuals with ASD in Türkiye have witnessed both progress and persistent challenges. Despite increased awareness and the availability of some standardized diagnostic tools, a comprehensive, nationwide framework for early identification and assessment remains lacking. The assessment process in Türkiye is typically initiated through referrals to child psychiatry services, where clinicians conduct evaluations based on developmental history, behavioral observations, and parent interviews. Practitioners in Türkiye frequently rely on translated or locally adapted instruments. A variety of screening and diagnostic tools for individuals with ASD have been translated, adapted, or developed for use in Türkiye. These instruments vary in terms of purpose (screening vs. diagnosis), age range, cultural adaptability, and psychometric robustness. Followings are the key tools that have been validated or widely utilized among Turkish populations:

The Rapid Interactive Screening Test for Autism in Toddlers (RITA-T) is a Level 2 interactive screening tool designed to identify early indicators of ASD in toddlers aged 18 to 36 months. It involves a series of structured play-based tasks administered by trained professionals. The RITA-T was psychometrically validated and demonstrated strong reliability and validity indicators [18]. The internal consistency of the instrument was high (Cronbach’s α = 0.89), and inter-rater reliability was excellent (Intraclass Correlation Coefficient [ICC] = 0.93), indicating consistent scoring across different evaluators. Test–retest reliability was also robust (r = 0.91), confirming the instrument’s stability over time. Concurrent validity was established through a strong correlation with the ADOS-2 (r = 0.87, *p* < 0.001), supporting the tool’s alignment with gold-standard diagnostic instruments. At the recommended cut-off score of ≥16, the RITA-T showed high sensitivity (91%) and specificity (89%), with a positive predictive value (PPV) of 86% and a negative predictive value (NPV) of 92%. Additionally, the area under the curve (AUC) from the ROC analysis was 0.94, indicating excellent discriminative ability. These findings affirm the Turkish version of RITA-T as a reliable and valid early screening tool for identifying toddlers at risk for ASD.

The Modified Checklist for Autism in Toddlers (M-CHAT) is a parent-report screening tool for toddlers aged 16 to 30 months. It is intended as a Level 1 screener to identify children at risk for ASD. The M-CHAT was examined and evaluated the applicability of the Turkish adaptation of the Modified Checklist for Autism in Toddlers, Revised with Follow-Up (M-CHAT-R/F) as a screening instrument within an urban, low-risk population of young children in Istanbul, Turkey [19]. A total of 6712 children aged between 16 and 36 months were screened using the M-CHAT-R/F. The Autism Diagnostic Observation Schedule-2 (ADOS-2) was employed as the primary diagnostic tool. Results indicated that 9.8% of the children screened positive on the initial M-CHAT-R/F. Of these, 39.7% were confirmed to meet criteria for Autism Spectrum Disorder (ASD) following the structured follow-up interview. In total, 57 children were diagnosed with ASD, corresponding to a prevalence rate of 0.8% (1 in 117; 95% CI: 0.063–1.05%). The findings demonstrated that the M-CHAT-R/F performed similarly in the Turkish context compared to its original use in the United States. M-CHAT remains a widely utilized screening instrument in pediatric and early education settings across Türkiye.

Childhood Autism Rating Scale (CARS), a widely used observational diagnostic tool, was psychometrically evaluated for Turkish populations [20]. In their validation study involving 199 children aged 18 to 97 months, CARS demonstrated high internal consistency (Cronbach’s α = 0.91), indicating excellent reliability across its 15-item structure. Inter-rater reliability was also strong, with Intraclass Correlation Coefficients (ICCs) exceeding 0.90, confirming the consistency of ratings across different clinicians. Concurrent validity was supported through significant correlations with DSM-5-based clinical diagnoses (r = 0.88, *p* < 0.001), and ROC analysis yielded an Area Under the Curve (AUC) of 0.95, suggesting excellent diagnostic accuracy. Sensitivity and specificity at the optimal cut-off point were reported as 90% and 92%, respectively, affirming CARS as an effective tool for distinguishing children with ASD from those with other developmental conditions in clinical settings.

The Turkish adaptation of the Gilliam Autism Rating Scale–Second Edition (GOBDÖ-2-TV) was conducted to assess the suitability of the instrument for use in Turkish clinical and educational contexts [21] and The Turkish Adapted Autism Behavior Checklist (U-ODKL), is a culturally tailored version of the original Autism Behavior Checklist (ABC) designed to assess autism-related behaviors in Turkish children [22]. Details on these scales are provided in Section 2.

Despite the availability of such tools, systemic challenges continue to impede early and accurate diagnosis. There is currently no mandatory developmental screening protocol in place in primary care settings, and general practitioners and pediatricians often lack sufficient training in recognizing early signs of ASD. These issues are exacerbated in rural and under-resourced regions, where access to specialized professionals and services is limited. Consequently, many children with ASD in Türkiye are diagnosed relatively late—often after entering primary school—thereby missing the critical window for early intervention. Sociocultural factors, including stigma and limited parental awareness, further delay the identification process. Research indicates that families frequently misinterpret early symptoms or hesitate to seek diagnostic services due to fear of labeling or discrimination. Despite improvements in ASD awareness and diagnosis globally, early detection in Türkiye remains a challenge due to limited access to trained professionals, delays in parental recognition, and disparities in screening availability across different regions. The Turkish government has implemented several policies and action plans to improve early diagnosis and intervention, including the National Action Plan for Autism Spectrum Disorder (2016–2019) and the more recent 2nd (2023–2028) National Action Plan [23]. To address these challenges, there is a growing consensus on the need for national policy reforms that integrate ASD screening into routine child health surveillance, strengthen intersectoral collaboration, and provide widespread training for frontline health and education professionals by UNICEF Türkiye [24]. In summary, while Türkiye has made strides in adapting ASD assessment tools and increasing public awareness, significant work remains in building a systematic and inclusive diagnostic infrastructure. Integrating validated, culturally sensitive instruments with early screening policies and equitable access to trained professionals will be essential for improving outcomes for individuals with ASD and their families nationwide.

Given the limitations of existing tools, there is a pressing need for a new, culturally adapted, and psychometrically validated autism assessment tool in Türkiye. A new assessment tool is needed to provide a standardized, easy-to-administer instrument that can be used by parents, educators and professionals, to address cultural and linguistic differences in ASD presentation among Turkish children, to offer robust psychometric properties, including strong validity and reliability measures, and to facilitate early and accurate identification of ASD to ensure timely intervention and support. By developing the ABAS, we aim to enhance the accessibility and accuracy of ASD assessment in Türkiye, ultimately improving diagnostic outcomes and intervention strategies for individuals with ASD. Moreover, tools like the ABAS, when supported by policy and professional development efforts, can contribute to a more equitable and effective assessment system across Türkiye.

### Purpose of the Study and Research Questions

The primary purpose of this study is to develop, validate, and standardize the Autism Behavior Assessment Scale (ABAS) (called Otizm Davranış Değerlendirme Aracı-ODDA in Turkish) as a culturally appropriate, psychometrically robust, and developmentally sensitive behavioral assessment tool for individuals diagnosed with or at risk for ASD in Türkiye. The ABAS aims to address the limitations of existing assessment instruments by offering a comprehensive, informant-based, and age-spanning measure suitable for both clinical and educational settings.

Based on the objectives outlined above, the study is guided by the following research questions:

1. What is the factorial structure of the ABAS and internal consistency and reliability across its subscales and total score?
2. Can the ABAS effectively differentiate between individuals diagnosed with ASD and those with other developmental conditions (i.e., intellectual disability, Down syndrome, or hearing impairment), indicating discriminant validity?
3. How well does the ABAS correlate with existing ASD diagnostic instruments, indicating convergent/criterion-related validity?
4. Is the ABAS stable over time, as evidenced by test–retest reliability?

## 2. Methods

### 2.1. Participants

This study involved a total of 1275 participants, including 1188 individuals diagnosed with ASD, 42 individuals diagnosed with intellectual disabilities (e.g., Down Syndrome), and 45 individuals diagnosed with hearing impairments. The data were collected from parents and professionals (e.g., educators, speech and language therapists, child development specialists, psychologists, psychological counselors, etc.) who had been working and had substantial knowledge of targeted individuals’ behaviors and social interactions across 14 provinces in Türkiye, including Ankara, Antalya, Bilecik, Bursa, Diyarbakır, Eskişehir, Istanbul, Izmir, Konya, Manisa, Mardin, Mersin, Muğla, and Şanlıurfa. The study aimed to ensure a broad geographic representation, enhancing the generalizability of the findings. The participants’ age range was between 3 years (36 months) and 24 years (288 months), with a mean age of 89 months (SD: 44.7). The inclusion criteria for individuals with ASD was that participants be diagnosed with ASD, as determined by child psychiatrists.

As shown in Table 1, the total sample included 1275 informants evenly divided between parents (*n* = 636) and educators/professionals (*n* = 639). The gender breakdown of the assessed individuals (*n* = 1275) shows a male predominance (75.4%, *n* = 961), which aligns with global prevalence data [1]. The sample primarily comprised individuals with ASD diagnosis (91.7%, *n* = 1170), but also included smaller groups with intellectual disabilities (3.7%, *n* = 48) and hearing impairments (4.6%, *n* = 58), supporting subsequent analyses of discriminant validity.

### 2.2. Data Collection

Prior to the psychometric analysis of the ABAS, ethical approval for the study was obtained. For this purpose, this study was conducted in accordance with the principles of the Declaration of Helsinki, and the research protocol was approved by the Ethics Committee of Anadolu University (Approval Code: 855642; Approval Date: 27 March 2025). A demographic information form and the ABAS items were digitized into a Google Form, and data were collected online. To recruit participants, contact was established with private special education and rehabilitation centers affiliated with the Turkish Ministry of National Education across various regions of Türkiye. Data were collected from parents and educators/professionals who volunteered to participate in the study of individuals diagnosed with autism. Informed consent was obtained from all participating parents and professionals prior to data collection, either electronically via the online Google form platform or through a hard copy. To ensure a broad geographic representation, enhancing the generalizability of the findings, participants were located in 14 provinces, including Ankara, Antalya, Bilecik, Bursa, Diyarbakır, Eskişehir, Istanbul, Izmir, Konya, Manisa, Mardin, Mersin, Muğla, and Şanlıurfa.

### 2.3. Instruments

The Autism Behavior Assessment Scale (ABAS) was developed in this study and The Turkish adaptation of the Gilliam Autism Rating Scale–Second Edition-GOBDÖ-2-TV [21] and The Turkish Adapted Autism Behavior Checklist-U-ODKL [22] were used to explore the criterion validity of the ABAS. In the following sections, GOBDÖ-2-TV and U-ODKL are explained, along with the development process for the ABAS.

The Turkish adaptation of the Gilliam Autism Rating Scale–Second Edition (GOBDÖ-2-TV). The GOBDÖ-2-TV was employed to assess the suitability of the instrument for use in Turkish clinical and educational contexts. In their validation study involving a large national sample of children aged 3 to 22 years, the GOBDÖ-2-TV demonstrated high internal consistency across its three subscales—Stereotyped Behaviors, Communication, and Social Interaction—with Cronbach’s alpha coefficients ranging from 0.88 to 0.92. Test–retest reliability was strong, with coefficients above 0.85 indicating stability over time. The instrument also showed robust inter-rater reliability (r = 0.87), supporting its consistency across different evaluators. Construct validity was confirmed through confirmatory factor analysis, which replicated the original three-factor structure. Criterion-related validity was established through significant correlations with clinical diagnoses of ASD and related behavioral measures.

The Turkish Adapted Autism Behavior Checklist (U-ODKL). The U-ODKL is a culturally tailored version of the original Autism Behavior Checklist (ABC) that is designed to assess autism-related behaviors in Turkish children. The validation study included a diverse sample of children diagnosed with ASD, and results indicated strong psychometric performance. The U-ODKL consists of 57 items across five domains: Sensory, Relating, Body and Object Use, Language, and Social and Self-Help. The instrument demonstrated high internal consistency, with a Cronbach’s alpha coefficient of 0.91 for the total scale. Subscale alphas ranged from 0.76 to 0.89, indicating adequate to strong reliability. Test–retest reliability, assessed over a two-week period, was r = 0.86, reflecting good temporal stability. Concurrent validity was supported through significant correlations with clinician-confirmed ASD diagnoses and other standardized measures. The tool also showed sensitivity of 88% and specificity of 85%, confirming its effectiveness in differentiating children with ASD from those with other developmental profiles. These results affirm the U-ODKL as a reliable and valid instrument for use in screening and assessing autism-related behaviors in various educational and clinical settings in Türkiye.

The Autism Behavior Assessment Scale (ABAS). To develop the ABAS, the following processes were carried out. This study followed a structured psychometric scale development process grounded in the three-phase, nine-step framework [25] ensuring the rigor and replicability of the Autism Behavior Assessment Scale (ABAS). The study integrates expert feedback, empirical data, and psychometric analyses to ensure that the scale is both theoretically sound and practically usable. The methodology encompassed comprehensive item generation, statistical validation, and standardization based on a large, heterogeneous sample drawn from multiple regions in Türkiye.

Initial items were developed through extensive literature review and alignment with DSM-5-TR criteria [1]. Behaviorally observable indicators across social interaction, communication, repetitive behaviors, and sensory sensitivities were emphasized. Expert panels comprising 12 field professionals (e.g., early interventionists, speech therapists, child psychiatrists) reviewed item relevance and cultural appropriateness. A total of 45 items were generated initially. After calculating the item-level content validity index (I-CVI), items with I-CVI < 0.80 were removed or revised. This process yielded 36 finalized items. Each item was rated on a 3-point Likert-type scale: 0 = Never Exhibits, 1 = Rarely/Sometimes Exhibits (2–3 times/day), and 2 = Frequently Exhibits (≥4 times/day). The final version of ABAS included four theoretically derived subscales: Restricted Repetitive Behaviors & Sensory Sensitivity (RRBSS)—9 items; Social Interaction (SI)—14 items; Social Communication (SC)—5 items; Non-Developmental Speech (NDS)—8 items (Please see Supplementary Materials-the ABAS Items). A Pilot version of the ABAS was administered to a small group of parents and professionals (*n* ≈ 30) to assess item clarity and cultural appropriateness. Feedback led to revisions in wording and item structure prior to large-scale administration. This pilot stage ensured face and content validity and informed the calculation of item-level content validity indices (I-CVIs), leading to the final 36-item version used in the main study.

To establish the psychometric robustness of the ABAS, construct validity, discriminant validity, criterion-related validity, internal consistency reliability, and test–retest reliability were explored, and normative data, cut-off classification, and ROC analysis were performed.

Exploratory Factor Analysis (EFA) was performed on parent data (*n* = 336) using Weighted Least Squares with Mean and Variance Adjusted (WLSMV) estimation in Mplus 8.3. Confirmatory Factor Analysis (CFA) was then conducted on the data from educators and professionals (e.g., speech and language therapists, child development specialists, psychologists, psychological counselors, etc.) (*n* = 336). Three models were tested: unidimensional, four-factor, and second-order.

To assess the ABAS’s ability to differentiate individuals with ASD from those with other developmental profiles, Welch’s ANOVA was used to compare total scores among three groups: individuals with ASD (*n* = 49), individuals with intellectual disabilities (ID; *n* = 42), and individuals with hearing impairments (HI; *n* = 45). These groups were selected because they often present with developmental characteristics that may overlap with ASD, such as communication delays, behavioral challenges, or sensory processing differences [9,11]). Including these comparison groups allowed us to test whether the ABAS could accurately differentiate ASD-specific behavioral profiles from other developmental conditions—an essential feature for any diagnostic tool. The 49 individuals with ASD used in the discriminant validity analysis were randomly selected from the full ASD sample (*N* = 1188) to match the age range of participants in the HI and ID groups. Selection prioritized individuals between 36 and 88 months old, enabling developmental comparability. The selection was stratified to maintain gender representation aligned with known ASD prevalence patterns [1]. Data were collected from 136 educators and professionals (speech and language therapists, child development specialists, psychologists, psychological counselors, etc.).

Criterion validity was evaluated by correlating the ABAS total scores with two widely used ASD instruments: the Turkish version of the Gilliam Autism Rating Scale–2 (GOBDÖ-2-TV) and the Turkish Adapted Autism Behavior Checklist (U-ODKL). Data were collected from 60 educators and professionals (speech and language therapists, child development specialists, psychologists, psychological counselors, etc.). Participants rated 60 individuals diagnosed with ASD using the ABAS, GOBDÖ-2-TV, and U-ODKL.

Internal consistency was evaluated using Cronbach’s alpha (α) and McDonald’s omega (ω).

Test–retest reliability was evaluated using Pearson’s r across two administrations of ABAS, conducted 15–20 days apart with a sub-sample of 307 educators and professionals (speech and language therapists, child development specialists, psychologists, psychological counselors, etc.).

To facilitate the clinical use of the ABAS, normative data and classification thresholds were developed. Raw scores were converted into standard scores and percentile ranks using the continuous norming method implemented in the cNorm Version 3.4.1 R package [26]. Age- and informant-specific norms were calculated but showed minimal variance, supporting the utility of unified cut-offs. ROC analysis informed the creation of risk classification levels for ABAS total scores, ranging from “No Risk” to “High Support Needs”. A raw score of ≥55 (85th percentile) was found to be the optimal threshold for indicating a high likelihood of ASD-related behavioral characteristics that warrant formal evaluation. These classification levels enable practitioners to interpret ABAS scores within a standardized framework and make informed, evidence-based decisions regarding screening, referral, or ongoing monitoring.

## 3. Results

### 3.1. The Construct Validity of the ABAS

Evidence regarding the construct validity of the ABAS has been presented based on factor analysis results. The 36 items included in the scale draft were first subjected to an Exploratory Factor Analysis (EFA) based on responses obtained from parents. To test the factor structure obtained from EFA, Confirmatory Factor Analysis (CFA) was conducted using data obtained from forms completed by educators. For both EFA and CFA, the WLSMV (Weighted Least Squares with Mean and Variance Adjusted) estimation method was employed, as it is appropriate for ordinal categorical data. Given the assumption that dimensions could be interrelated, an oblique rotation method was utilized in EFA.

Factor Structure Expectation and Missing Data Handling. The scale was theoretically expected to exhibit a three-dimensional structure. Accordingly, the Restricted Repetitive Behaviors & Sensory Sensitivity (RRBSS) subscale was designed to include 9 items, the Social Interaction & Communication (SI) subscale to include 19 items, and the Non-Developmental Speech (NDS) subscale to include 8 items. Notably, the final eight items were designed only for verbal individuals, meaning that responses for non-verbal individuals were coded as 0 (not exhibiting the behavior). The percentage of missing data among the 36 items was 0.01. As missing values were minimal and determined to be randomly distributed, a single imputation method was employed using chained equations, taking into account the ordinal categorical nature of the data. The “mice version 1.0” package in R [27]. was utilized for this imputation process. Following imputation, the dataset was divided into two groups: 336 parents (for EFA) and 336 professionals (for CFA). EFA and CFA analyses were conducted using Mplus 8.3 [28]. Internal consistency reliability was assessed using the “psych” and “sirt” packages in R.

Factor Analysis Results and Model Fit. Although the scale was theoretically expected to be three-dimensional, factor analyses were conducted for models ranging from one to five dimensions. The model-data fit indices and the theoretical interpretability of each dimensional solution were evaluated. The fit indices for one- to five-factor models are presented in Table 2.

Root Mean Square Error of Approximation (RMSEA) and Standardized Root Mean Square Residual (SRMR) values below 0.05 indicate good model-data fit, while values between 0.05 and 0.08 suggest an acceptable model-data fit. Similarly, Comparative Fit Index (CFI) and Tucker–Lewis Index (TLI) values greater than 0.95 indicate good model-data fit, whereas values between 0.90 and 0.95 suggest an acceptable fit. Accordingly, the one- and two-factor EFA models yielded poor model-data fit indices. The three-factor solution, which aligns with the theoretical structure, demonstrated acceptable model-data fit values, while the four- and five-factor solutions indicated good model-data fit. Upon examining the factor loadings of the three-factor structure, it was found that the item distribution did not align with theoretical expectations, with multiple cross-loading items and unclear item distributions across dimensions. Conversely, for the four- and five-factor models, the factor distributions were more theoretically interpretable.

Among these, the four-factor model was determined to be the most theoretically appropriate and interpretable solution. In this structure, the nine-item Restricted Repetitive Behaviors & Sensory Sensitivity (RRBSS) subscale and the eight-item Non-Developmental Speech (NDS) subscale maintained their integrity. Additionally, the original 19-item Social Interaction & Communication (SI) subscale was divided into two separate dimensions: 14 items forming the Social Interaction (SI) subscale and 5 items forming the Social Communication (SC) subscale. As a result, the final four-factor structure was deemed theoretically sound and interpretable. This model accounts for 56% of the total variance.

The factor loadings of the 36 items in the ABAS across four dimensions are presented in the Table 3. A factor loading threshold of 0.40 is recommended to determine whether an item is sufficiently related to its respective dimension.

Among the items in the scale, only item 17 had a slightly lower factor loading (0.37). However, given that a widely accepted alternative threshold is 0.32, this value is still considered acceptable. If an item was associated with multiple dimensions with a factor loading of 0.32 or higher, it was assigned to the dimension with the strongest loading. In such cases, a minimum difference of 0.10 between factor loadings across dimensions was required for item placement. All items met this criterion, as each item demonstrated the strongest association with its theoretically expected dimension, with a difference of at least 0.10 from factor loadings on other dimensions. Therefore, all items were retained in the final version of the scale, as their factor loadings and theoretical relevance to their designated dimensions were deemed appropriate.

Finally, the internal consistency reliability of the final scale was assessed following EFA implementation. Cronbach’s alpha (α) and McDonald’s omega (ω) coefficients were calculated for each dimension, while a stratified alpha was reported for the entire scale. The results are presented in Table 4.

The results indicate that the Cronbach’s alpha (α) values for the subscales range between 0.78 and 0.86, while McDonald’s omega (ω) values range between 0.84 and 0.90. The internal consistency reliability for the entire scale was calculated as 0.91. These values suggest that the measurements obtained using the ABAS exhibit high internal consistency reliability.

In the EFA implementation, data collected from parents were used to analyze the ABAS’s factor structure. To validate the four-factor structure identified in the EFA, a Confirmatory Factor Analysis (CFA) was conducted using 336 observations from teachers. CFA is used to test the theoretical factor structure of a measurement tool. Based on the EFA results, items were assigned to their expected dimensions, and CFA was applied.

As in EFA, the WLSMV (Weighted Least Squares with Mean and Variance Adjusted) estimation method was used in CFA due to the ordinal categorical nature of the data. Although the expected structure was multidimensional, the principle of parsimony suggests that unidimensionality should be tested first in CFA applications. After evaluating the one-factor model, the four-factor correlated model was tested. Finally, since there was a need to obtain an overall score from the scale, a second-order CFA was conducted, and its fit was reported as well. The model-data fit indices for these three CFA models are presented in Table 5.

**Figure 1:**
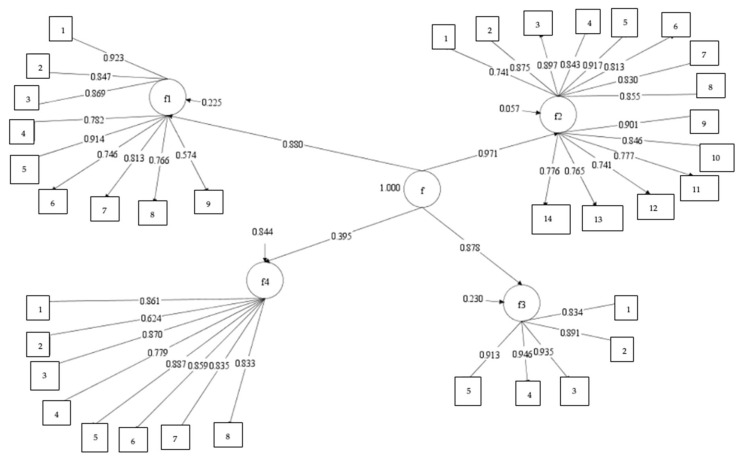
Estimates obtained for the second-order correlated factors model. (Note. f: factor).

Upon examining the values in Table 5, it is evident that the one-factor model exhibits poor model-data fit. In contrast, the values obtained for the theoretically expected four-factor model indicate an acceptable fit for SRMR, while the remaining indices suggest a good model fit. Finally, an examination of the second-order CFA model fit reveals that its fit indices are slightly better than those obtained for the four-factor model. In other words, both the four-factor correlated model and the second-order CFA model demonstrate a strong fit to the data. The factor loadings and correlations between the dimensions in the second-order four-factor correlated model are presented in Figure 1.

Upon examining the obtained factor loadings, it is observed that all loadings are 0.50 or above. Furthermore, 34 of the items have factor loadings above 0.70. These findings suggest that the factor analytic structure derived from data collected through the parent-rated version of the ABAS is confirmed by the educator/professional-rated form. Lastly, the internal consistency reliability of the dataset used in the Confirmatory Factor Analysis (CFA) was assessed. As in the earlier analyses, Cronbach’s alpha and McDonald’s omega coefficients were calculated for each dimension, and stratified alpha was calculated for the overall scale. The results are presented in Table 6.

As shown in Table 6, Cronbach’s alpha values range from 0.89 to 0.94 across the dimensions, while McDonald’s omega values range from 0.92 to 0.95. The stratified alpha for the total scale is 0.96. These findings indicate that the internal consistency of the educator/professional-rated ABAS data is very high. The results also demonstrate that the scale exhibits a theoretically interpretable four-factor structure and that the structure identified from parent-reported data is strongly confirmed with educator/professional-rated data. Consequently, the construct validity of the ABAS is considered to be very strong.

### 3.2. The Discriminant Validity of the ABAS

To assess the discriminant validity of the ABAS, the scale was completed by educator/professionals for a total of 108 participants who had a medical diagnosis of ASD (ASD, *n* = 49), intellectual disability/Down syndrome (ID/DS; *n* = 42), or hearing impairment (HI; *n* = 45). Descriptive findings related to the gender variable of the assessed individuals are presented in Table 7. In terms of age, the participants with ASD ranged between 36 and 88 months (mean age = 42 months), those with intellectual disability/Down syndrome (ID/DS) between 36 and 78 months (mean age = 44 months), and those with hearing impairment (HI) between 36 and 86 months (mean age = 46 months).

As shown in Table 8 and Table 9, the results of the One-Way ANOVA (Welch’s test) indicate that there is a statistically significant difference between the ABAS scores of individuals with ASD, intellectual disability/Down syndrome, and hearing impairment.

The results of the Tukey Post Hoc Test (Table 10), conducted to identify between-group differences, show that the ABAS significantly differentiates individuals with ASD from those in the other two groups. These findings provide strong evidence for the high discriminant validity of the ABAS.

Receiver Operating Characteristic (ROC) Analysis Findings. We employed a Receiver Operating Characteristic (ROC) analysis to evaluate the diagnostic performance of the ABAS in distinguishing individuals with AS from those with other disabilities, intellectual disability/Down syndrome, and hearing impairments. The analysis included data from 134 participants (ASD = 46, DS = 43, Hearing = 45). The findings revealed a sensitivity of 97.06% and a specificity of 98.77% for a cut-off score of 4. The area under the ROC curve (AUC) was 0.99, indicating excellent discriminative ability. These results suggest that the ABAS is a highly reliable tool for identifying individuals with ASD when using the specified cut-off score, providing strong support for its use as a screening or diagnostic tool in educational and clinical settings. The high Youden’s index further confirms the balance between sensitivity and specificity at the selected threshold.

### 3.3. The Criterion-Related Validity of the ABAS

To evaluate the criterion-related validity of the ABAS, two instruments with established validity and reliability in Türkiye were used: the Gilliam Autism Rating Scale–Second Edition, Turkish Version and the Turkish Adapted Autism Behavior. The autism-related behaviors of 60 children diagnosed with ASD—48 boys and 12 girls, aged between 36 and 163 months (mean age = 79 months)—were assessed by educators/professionals using the ABAS, GARS-2-TV, and U-ODKL forms. Data were analyzed using Pearson correlation coefficients in the Jamovi statistical software (Version 2.5.3.0). Based on the ABAS norm score evaluations, the correlation between the ABAS norm scores and GARS-2-TV norm scores was r = 0.93 (*p* < 0.001), and the correlation between the ABAS norm scores and U-ODKL norm scores was r = 0.84 (*p* < 0.001). These findings indicate a strong positive correlation, supporting the high criterion-related validity of the ABAS.

### 3.4. The Test-Retest Reliability of the ABAS

To assess the test–retest reliability of the ABAS, data were collected from 307 educators/ professionals at two time points 15–20 days apart. Each participant assessed the same child with a confirmed ASD diagnosis at both time points. The children assessed were aged between 36 and 188 months, with a mean age of 78 months. The sample included 83 girls and 224 boys. Based on norm score evaluations, the Pearson correlation coefficient between the first and second assessments was r = 0.83 (*p* < 0.001), indicating a strong positive correlation. This result suggests that the ABAS has high test–retest reliability.

## 4. Discussion and Conclusions

The present study aimed to develop, validate, and standardize the Autism Behavior Assessment Scale (ABAS), a culturally adapted and developmentally appropriate tool designed to assess autism-related behaviors in individuals aged 3 to 24 years in Türkiye. Rooted in a robust psychometric framework [25], the development of the ABAS addressed a critical need within the Turkish context for a reliable and accessible measure that reflects both local cultural–linguistic norms and international standards of autism assessment. The ABAS was constructed de novo, rather than translated or adapted from existing tools, which allowed the research team to ensure alignment with Türkiye’s sociocultural context, educational settings, and clinical realities.

### 4.1. Summary and Interpretation of Key Findings

The ABAS demonstrated strong psychometric properties across all phases of the validation process. Exploratory and confirmatory factor analyses supported a four-factor model encompassing Restricted Repetitive Behaviors & Sensory Sensitivity (RRBSS), Social Interaction (SI), Social Communication (SC), and Non-Developmental Speech (NDS). This model is conceptually consistent with the two core diagnostic criteria outlined in the DSM-5-TR [1] and provides a nuanced representation of the behavioral phenotype of autism across different age groups and functional levels.

The findings underscore the ABAS’s capacity to serve as both a diagnostic adjunct and a tool for educational and clinical decision-making in Türkiye, where standardized assessments like ADOS-2 are often inaccessible. The statistically robust structure of the ABAS—grounded in both empirical validation and cultural sensitivity—demonstrates the feasibility of constructing psychometrically sound tools within non-Western contexts, a significant advancement in the global landscape of autism assessment [2,29].

### 4.2. Cultural and Cross-Contextual Considerations

Cultural sensitivity in autism assessment is essential to prevent underdiagnosis, misdiagnosis, or inappropriate intervention planning. In Türkiye, widespread disparities in access to gold-standard diagnostic tools such as ADOS-2 or ADI-R are compounded by the uneven distribution of trained professionals, regional socioeconomic differences, and varying parental awareness levels []. These challenges create a landscape in which behavioral checklists—especially those that are easy to administer and interpret—become critical tools in identifying children at risk for ASD.

The ABAS addresses this gap by providing an instrument that not only reflects Turkish cultural and linguistic norms but also meets rigorous international validation standards. While no specific cross-cultural adaptation was needed (since the tool was developed in Turkish for a Turkish population), careful attention was paid to ensuring that item phrasing was free of cultural or linguistic bias and that behaviors were observable in naturalistic Turkish social contexts (e.g., school, family, clinic). Moreover, the use of both parent and professional informants helps mitigate bias by integrating multiple perspectives.

As cultural perceptions of disability and neurodiversity evolve in Türkiye, tools like the ABAS have the potential to promote early identification and inclusive education practices. This aligns with the goals of the National Autism Action Plan (2023–2028) and recommendations by international organizations such as UNICEF Türkiye (2023), which advocate for contextually grounded assessment systems.

The successful validation of a locally developed tool like ABAS reinforces the argument that culturally tailored instruments—when developed with methodological rigor—can produce reliable and generalizable results within their context. This contrasts with the common but problematic reliance on direct translations of Western tools that may not align with local norms [10].

### 4.3. Contributions to the Field

This study provides multiple theoretical, methodological, and applied contributions to the field of autism assessment and early intervention.

Theoretical advancement: The ABAS confirms that autism-related behaviors can be reliably observed and differentiated across diverse contexts and informants using a multi-dimensional model that aligns with established diagnostic frameworks.

Methodological innovation: Unlike many existing measures, the ABAS was developed using a three-phase, nine-step model for scale development [25] and subjected to rigorous content validation procedures, including item-level content validity index (I-CVI), Scale-level CVI, and Cohen’s kappa.

Cultural specificity: Developed and normed entirely in Türkiye, the ABAS is sensitive to local communication styles, social expectations, and professional practices—factors critical to both its ecological validity and user adoption.

Practical application: With age-specific but unified norms, cut-off scores based on ROC analysis, and informant flexibility, the ABAS is designed for use by educators, clinicians, and researchers in a wide range of settings—from special education classrooms to early childhood centers and community-based intervention programs.

In sum, the utility of the ABAS extends beyond statistical significance. Its application can directly address systemic barriers in Türkiye’s diagnostic pathways—such as long wait times, insufficient clinician training, and geographic disparities in service access. By offering a scalable and culturally congruent assessment alternative, the ABAS may reduce the average age of ASD diagnosis and increase equity in access to early intervention services [24].

### 4.4. Limitations and Methodological Considerations

Despite its strengths, this study is not without limitations. First, while the sample was large (*N* = 1275) and geographically diverse (across 14 provinces), recruitment relied on purposive and convenience sampling through special education centers, potentially limiting representativeness, especially from rural or under-resourced regions. Second, although the use of multiple informants (parents and professionals) enhanced ecological validity, the reliance on informant reports introduces the potential for bias due to subjective interpretation of behaviors. Differences in training, familiarity with ASD, or cultural expectations may influence scoring patterns. Third, although diagnostic status was verified through documentation from licensed child psychiatrists, no standardized severity-level classification (e.g., mild/moderate/severe ASD or ID) was available. Future versions of the ABAS may benefit from stratifying results based on functional profiles or co-occurring conditions. Fourth, the study was cross-sectional in design. While test–retest reliability was assessed, the scale’s sensitivity to developmental changes or intervention effects remains unknown. Longitudinal studies will be essential to determine the ABAS’s utility in monitoring behavior over time. Finally, while ABAS showed strong criterion validity with established Turkish tools, comparison with international gold standards such as ADOS-2 or ADI-R was not possible due to availability limitations. This limits the ability to directly benchmark the ABAS against widely accepted diagnostic procedures.

### 4.5. Implications for Practice, Research, and Policy

The ABAS provides an empirically validated, culturally appropriate framework for behavioral screening of ASD in Türkiye. It can support early detection in preschool and early school years, differential identification of ASD vs. ID or HI in inclusive classrooms, progress monitoring in special education or rehabilitation settings, and family education and consultation, especially in under-resourced areas. In clinical settings, the ABAS offers a pragmatic alternative to time-intensive diagnostic interviews. For educational professionals, it enables structured observations that can inform Individualized Education Plans (IEPs) and transition support. Policymakers may also consider its integration into national early childhood screening systems, contributing to a more equitable ASD identification framework. For researchers, the ABAS can facilitate longitudinal studies, group comparisons, and culturally sensitive evaluations of intervention efficacy. Its open-access design and statistical transparency make it highly adaptable for further psychometric refinement.

### 4.6. Future Directions

To extend the utility of the ABAS, future research should aim to validate the tool across larger, stratified, and nationally representative samples, including underserved rural and minority populations; examine its performance in multilingual contexts or among Turkish-speaking communities abroad (e.g., Germany, Northern Cyprus); develop a digital scoring and reporting platform to streamline implementation and increase accessibility; conduct longitudinal studies to assess sensitivity to developmental progression and behavioral change in response to intervention; investigate measurement invariance across informants, genders, and age groups to determine if separate norms or cutoffs are needed; and expand the ABAS to include additional domains such as emotional regulation, executive function, or adaptive behavior, aligning with emerging models of ASD heterogeneity and developmental comorbidity.

## 5. Conclusions

The ABAS is a significant advancement in autism assessment practices in Türkiye. By combining cultural specificity with rigorous psychometric standards, it offers a reliable, valid, and accessible tool for behavioral screening, particularly in contexts where traditional diagnostic systems are not fully accessible. The ABAS not only contributes to the scientific literature on scale development but also has immediate implications for practice, policy, and family support systems in the field of developmental disabilities. Ultimately, the ABAS exemplifies how locally developed tools—when grounded in empirical rigor and cultural relevance—can contribute to more inclusive, timely, and effective support systems for individuals with autism and their families.

Overall, this study not only addresses a critical gap in Türkiye’s autism assessment infrastructure but also contributes to the broader field of culturally responsive psychometrics. The ABAS is positioned to become an integral component of early screening, diagnosis, and educational planning, with the potential for adaptation in other Turkish-speaking populations globally.

## Figures and Tables

**Table 1:** Demographic characteristics of the sample (with frequencies).

Variable	Parents (*n* = 636)	Educators/Professionals (*n* = 639)	Total (*N* = 1275)
Child Age (months)			
– Mean (SD)	87.4 (43.9)	90.2 (45.3)	89.0 (44.7)
– Range	36–288	36–288	36–288
Child Gender			
– Male	485 (76.2%)	476 (74.5%)	961 (75.4%)
– Female	151 (23.8%)	163 (25.5%)	314 (24.6%)
Diagnostic Category			
– Autism Spectrum Disorder	591 (92.8%)	579 (90.5%)	1170 (91.7%)
– Intellectual Disability (Down Syndrome)	23 (3.6%)	25 (3.9%)	48 (3.7%)
– Hearing Impairment	23 (3.6%)	35 (5.6%)	58 (4.6%)

Note: Frequencies are followed by percentages in parentheses.

**Table 2:** Fit indices obtained from EFA for 1 to 5 factors.

Number of Factors	Fit Indices			
	RMSEA	CFI	TLI	SRMR
1	0.084 (0.080–0.088)	0.81	0.80	0.123
2	0.064 (0.059–0.068)	0.89	0.88	0.086
3	0.050 (0.045–0.055)	0.94	0.93	0.066
4	0.038 (0.032–0.044)	0.97	0.96	0.052
5	0.026 (0.018–0.033)	0.99	0.98	0.042

Note: 1. The eigenvalue for Factor 1 = 11.898, Factor 2 = 3.853, Factor 3 = 2.438, Factor 4 = 1.975, and Factor 5 = 1.421.

**Table 3:** Standardized factor loadings for the four-factor EFA model.

Items	Factor 1 (RRBSS)	Factor 2 (SI)	Factor 3 (SC)	Factor 4 (NDS)
1	0.682 *			
2	0.676 *			
3	0.691 *			
4	0.691 *			
5	0.670 *			
6	0.572 *			
7	0.672 *			
8	0.566 *			
9	0.444 *			
10		0.414 *		
11		0.635 *		
12		0.702 *		
13		0.727 *		
14		0.704 *		
15		0.545 *		
16		0.589 *		
17		0.369 *		
18		0.503 *		
19		0.445 *		
20		0.596 *		
21		0.634 *		
22		0.611 *		
23			0.764 *	
24			0.708 *	
25			0.860 *	
26			0.566 *	
27		0.640 *		
28			0.551 *	
29				0.678 *
30				0.660 *
31				0.767 *
32				0.605 *
33				0.545 *
34				0.644 *
35				0.725 *
36				0.515 *

Note: r_f1,f2_ = 0.488 *, r_f1,f3_ = 0.387 *, r_f1,f4_ = 0.134, r_f2,f3_ = 0.435 *, r_f2,f4_ = 0.116, ve r_f3,f4_ = 0.248. * *p* < 0.05.

**Table 4:** Internal consistency coefficients of the ABAS based on EFA results.

Internal Consistency	Factor 1 (RRBSS)	Factor 2 (SI)	Factor 3 (SC)	Factor 4 (NDS)
α (Cronbach’s Alpha)	0.86	0.88	0.83	0.78
ω (McDonald’s Omega)	0.88	0.90	0.86	0.84
Stratified (Total) α	0.91			

**Table 5:** Model fit indices for Confirmatory Factor Analysis (CFA) of the ABAS.

Model	Fit Indices			
	RMSEA	CFI	TLI	SRMR
Unidimensional	0.122 (0.118–0.126)	0.85	0.84	0.143
Four-Factor	0.050 (0.045–0.054)	0.98	0.97	0.067
Second-Order	0.048 (0.044–0.053)	0.98	0.97	0.068

**Table 6:** Internal consistency coefficients of the ABAS based on CFA results.

Internal Consistency Coefficient	Factor 1 (RRBSS)	Factor 2 (SI)	Factor 3 (SC)	Factor 4 (NDS)
α (Cronbach’s Alpha)	0.90	0.94	0.91	0.89
ω (McDonald’s Omega)	0.93	0.95	0.94	0.92
Stratified (Total) α	0.96			

**Table 7:** Descriptive results.

Gender	Diagnosis	n	%	Cumulative %
Female	ASD	10	7.4 %	7.4 %
	ID/DS	12	8.8 %	16.2 %
	HI	16	11.8 %	27.9 %
Male	ASD	39	28.7 %	56.6 %
	ID/DS	30	22.1 %	78.7 %
	HI	29	21.3 %	100.0 %

**Table 8:** Descriptive statistics of groups.

	Diagnosis	N	X	SS
ABAS Norm Score	ASD	49	104.8	13.90
	ID/DS	42	74.5	4.10
	HI	45	78.7	1.52

**Table 9:** One-Way ANOVA (Welch’s) results.

	F	df1	df2	*p*
ABAS Norm Score	108	2	67.7	<0.001

**Table 10:** Tukey Post Hoc Test results.

Comparison	Mean Difference	*t*-Value	df	*p*-Value
ASD vs. ID/DS	30.4	16.7	134	<0.001
ASD vs. HI	26.15	14.60	134	<0.001
ID/DS vs. HI	−4.20	−2.27	134	0.063

## Data Availability

The data presented in this study are available on request from the corresponding author due to ethical reasons.

## Supplementary Materials

The following supporting information can be downloaded at: https://www.mdpi.com/article/10.3390/children12081038/s1, the ABAS Items.
