# Supplementary material for: Development and Validation of the Autism Behavior Assessment Scale (ABAS)
Source: Children (Basel). 2025 Aug 8;12(8):1038. doi: 10.3390/children12081038 (PMC12384625; doi:10.3390/children12081038)
Supplement: Supplementary file 1 [file children-12-01038-s001.zip › children-3759333-supplementary.pdf]

Supplemental File:

Autism Behavior Assessment Scale (ABAS) Items

Instructions for Respondents. This scale is designed to assess observable behaviors typically associated with Autism Spectrum Disorder (ASD). It should be completed by someone who regularly interacts with the child (e.g., parent, teacher, caregiver, professional) and knows the child well. Rate each item based on how often the behavior occurs during a typical day, not based on past behavior or one-time situations. Consider whether the behavior is developmentally appropriate or whether it reflects a pattern that is restricted, repetitive, or qualitatively different from age-appropriate development.
Rating Scale: 0 = Never: Behavior is not observed during the day, **1 = Sometimes**: Behavior is observed 2–3 times per day, **2 = Frequently**: Behavior is observed 4 or more times per day.

Subscale 1: Restricted Repetitive Behaviors & Sensory Sensitivity (RRBSS)
1. When alone, spends most of his/her time engaged in repetitive and restrictive behaviors.
Explanation: For example, aimlessly swaying side to side or back and forth, frequently twisting hands at eye level, flicking fingers, repeatedly hitting the head or body, aimlessly waving hands, aimlessly flapping hands, spinning around, etc.
2. Shows an obsessive level of interest in specific objects or toys.
Explanation: For example, excessive interest in a shiny object, a scented eraser, a spinning material, velvet fabric, a toy car, or a part of a toy (carrying it in their hand or pocket, constant desire to engage with the same thing).
3. Produces incomprehensible sounds and repeats them repeatedly.
Explanation: For example, it makes sounds like "eee-eeeee-eee" or "iiii-iiii-iiii."
4. Has specific routines/rituals and repetitive behavior patterns.
Explanation: For example, touches certain places in a specific order when entering a room, insists on taking the same route to school. May have rituals related to dressing and undressing; for example, insists on wearing clothes in a specific color or order, etc.
5. Displays aimless, inappropriate repetitive behaviors while playing with objects or toys.
Explanation: For example, arranges objects or toys side by side or in a row; scrapes cars on the ground to make them go faster and repeats this behavior continuously without a play purpose, to self-stimulate, listens to the sound produced, watches the wheels turn, etc.
6. Sniffs, tastes, sucks, or tries to eat non-renewable objects.
Explanation: For example, sniffs, tastes, sucks, or tries to eat objects such as human hands, toys, books, scented erasers, fabric, or hair.
7. Gives unusual responses to sensory inputs.
Explanation: For example, gives different responses to pain/heat/cold/certain sounds/textures or surfaces. Excessively smells or touches certain objects. Reacts to lights, sounds, or moving objects as if mesmerized or highly distressed (e.g., covering ears, closing eyes, screaming, pretending not to hear, hitting oneself, freezing, etc.).
8. When something goes wrong or routines change, they need a lot of reassurance.
Explanation: For example, when they come home from outside and see that their belongings have been moved around or some of them have been taken out of their room, when their weekend breakfast routine is canceled, or when their favorite meal or weekly visit on Sundays is changed, they become upset, restless, unable to tolerate it, and feel a strong need for comfort.
9. Shows unusual food selectivity.
Explanation: For example, insists on eating the same type of food consistently based on whether the food is soft, pureed, or hard, overly sweet, sour, or salty, only hot or cold, or based on its color or smell.

Subscale 2: Social Interaction (SI)

10. Avoids eye contact or makes brief/short eye contact.

Explanation: Does not look at the face of the person trying to communicate with them, looks away when they do look at them. Avoids eye contact or makes brief/short-lived eye contact.

11. Does not initiate social interaction with peers or other people.

Explanation: Does not initiate communication with the person in front of them for the purpose of social interaction. Does not make communication attempts to express requests, share interests, excitement, opinions, etc. Does not make efforts to play with other people at school, in the park, etc., or to approach them for the purpose of communication, such as showing interest or curiosity, does not go to them, or make small talk.

12. Pays little or no attention to what peers are doing.

Explanation: For example, when a peer is filling a truck with a shovel in the sandbox at the park, they only pay attention for a moment or do not show any interest. They do not engage in games such as building with Legos or playing house with toys at the nursery, do not go over to their peers to watch with interest, or follow them with curiosity. Even if they glance briefly, they are more preoccupied with their own interests.

13. Is unsuccessful in imitating others in learning activities or games.

Explanation: For example, in games and educational activities prepared by the teacher for the class, the child does not try to perform the activity by imitating the teacher or a friend. When the parent/sibling/peer is rocking a doll, serving tea with a toy teapot, stacking nested buckets/rings to build a tower, etc., the child does not attempt to imitate these actions.

14. Does not pay attention to or care about others' attention or interest.

Explanation: For example, does not pay attention to what others (peers, parents, adults) do to get attention (showing toys, acting spoiled/playing, sticking out tongue, hand/arm movements, etc.), and does not show any response (smiling, blushing, etc.) to such attention.

15. Does not try to get someone else's attention directly.

Explanation: For example, does not show objects or toys to get someone's attention or engage in attention-seeking behaviors/games (such as sticking out the tongue, hand gestures, etc.).

16. Shows very little satisfaction when interacting with others.

Explanation: For example, when someone tries to talk to them, join their game, etc., they do not show satisfaction by smiling, cooperating, participating in what is being done, etc., due to the other person's presence, actions, or words.

17. Shows more interest in objects than in people.

Explanation: For example, instead of interacting with people in the environment (acting indifferently as if no one else is there), spends time with objects or toys that interest them.

18. Has difficulty establishing reciprocal social interaction.

Explanation: When someone initiates communication with them, for example by asking how they are, they do not respond or take turns to maintain continuity in the interaction.

19. Does not exhibit symbolic or imaginary play behaviors.

Explanation: For example, does not pretend to use a remote control as a phone, use a shoe box as a garage, create a village or zoo with animals or Legos, or engage in games such as house or doctor play, nor can they play these games.

20. Does not look at the person who says this/her name when it is spoken.

Explanation: For example, during group interactions or one-on-one interactions, they occasionally respond to their name, but these responses are inconsistent, and they often do not turn around.

21. Does not express a desire for what s/he wants or gives up easily.

Explanation: For example, does not make verbal requests (give, baby, car, etc.), gestures or facial expressions (pointing, reaching, etc.), or vocalizations (uh-uh, etc.) for what they want, or does not persist in such attempts.

22. Gives inappropriate responses to humorous or comical situations.
Explanation: For example, does not laugh at funny stories, cartoons, jokes, or humor, does not find humor in comical situations such as someone being clumsy (falling, acting like a clown, etc.), does not respond, or may respond inappropriately (become angry, etc.).
23. Does not point with a finger in appropriate situations.
Explanation: For example, does not point with a finger to indicate something they want, expect, find interesting, see for the first time, etc., or to show someone else something they are looking for, etc.

Subscale 3: Social Communication (SC)
24. Has difficulty understanding expressions such as slang, metaphors, idioms, and proverbs.
Explanation: For example, the phrase "my two feet are in one shoe" can be thought of as "being in a hurry instead of having both feet in one shoe," "my head is as big as a pot" could be interpreted as "the head is like a pot," "the head has grown," or "I'm so hungry I could eat you."
25. Has difficulty understanding when someone is teasing, provoking, mocking, or flattering them.
Explanation: For example, they have difficulty understanding when someone is deliberately trying to provoke them, using various hints or words to target them, or when someone is mocking them, teasing them, or saying positive things to them using various hints or words.
26. **Does not appear to understand that other people have feelings and thoughts that are different from their own.**
Explanation: For example, they cannot consider that not everyone likes ice cream, that some people may like sour/bitter tastes, or that some people may prefer reading books to watching television.
27. Has difficulty understanding other people's body language.
Explanation: For example, has difficulty understanding when someone else is sad based on their gestures and facial expressions. Has difficulty understanding when someone else does not want to communicate with them (e.g., looking away, turning their back, talking to someone else, or avoiding eye contact).
28. Has difficulty recognizing and interpreting others' reactions.
Explanation: Has difficulty interpreting the reactions of others, can only interpret superficial reasons. For example, cannot understand that someone does not want to go to the park with them because they are angry or upset.

Subscale 4: Non-Developmental Speech (NDS)
29. Repeats words and phrases immediately or later.
Explanation: For example, when asked "Hello, how are you?" instead of saying "I'm fine," they repeat the exact phrase they heard, such as "Hello, how are you?" or say "Be a flower" out of the blue, or repeat a phrase heard from a vendor at the market or a movie line.
30. Prefers to talk obsessively about a fixed area of interest.
Explanation: For example, they want to talk endlessly about a specific topic they are interested in (insects, trains, car brands, dinosaurs, computers, etc.).
31. Uses words or phrases that are out of context.
Explanation: Uses words or expressions unrelated to the conversation during a conversation. For example, during a conversation, they may use expressions such as "ice cream," "gooool," "50% discount," or "buy one, get one free," which are unrelated to the topic.
32. Speaks in a flat tone that does not reflect emotion.
Explanation: For example, even if the topic is exciting, emotional, or surprising, they speak in a monotonous, unchanging manner without reflecting their emotions on the topic they are discussing. They do not use emphasis to highlight points they want to emphasize in their sentences, do not use question intonation when asking questions, or use question intonation in all their sentences, and may speak in a very low or very high voice, etc.

33. Uses the words "yes" and "no" inappropriately.
Explanation: For example, when asked if they want something they like or an activity, they may say "No"; when asked about something they dislike or an activity, they may say "Yes."
34. When talking about themselves, they use the third-person singular pronoun "he/she" or their name instead of "I."
35. Uses made-up words or phrases that have no meaning.
36. Does not initiate conversation with peers or adults.
Explanation: For example, does not make small talk or attempt to start a conversation with peers or adults due to reasons such as interest or need.
